# A beneficial role of computer-aided diagnosis system for less experienced physicians in the diagnosis of thyroid nodule on ultrasound

**DOI:** 10.1038/s41598-021-99983-6

**Published:** 2021-10-14

**Authors:** Sunyoung Kang, Eunjung Lee, Chae Won Chung, Han Na Jang, Joon Ho Moon, Yujin Shin, Kyuho Kim, Ying Li, Soo Myoung Shin, Yoo Hyung Kim, Seul Ki Kwon, Chang Ho Ahn, Kyong Yeun Jung, A. Ram Hong, Young Joo Park, Do Joon Park, Jin Young Kwak, Sun Wook Cho

**Affiliations:** 1 Department of Internal Medicine, Seoul National University College of Medicine, Seoul, Republic of Korea; 2 Department of Internal Medicine, Seoul National University Hospital, Seoul, Republic of Korea; 3 Department of Internal Medicine, Uijeongbu Eulji Medical Center, Eulji University School of Medicine, Uijeongbu-si, Republic of Korea; 4 School of Mathematics and Computing, Yonsei University, Seoul, Republic of Korea; 5 Department of Internal Medicine, Seoul National University Bundang Hospital, Seongnam, Republic of Korea; 6 Department of Internal Medicine, Nowon Eulji Medical Center, Eulji University School of Medicine, Seoul, Republic of Korea; 7 Department of Internal Medicine, Chonnam National University Medical School, Gwangju, South Korea; 8 Department of Molecular Medicine and Biopharmaceutical Sciences, Graduate School of Convergence Science and Technology, Seoul National University, Seoul, Republic of Korea; 9 Department of Radiology, Research Institute of Radiological Science, Yonsei University College of Medicine, Seoul, Republic of Korea

**Keywords:** Cancer, Endocrinology

## Abstract

Ultrasonography (US) is the primary diagnostic tool for thyroid nodules, while the accuracy is operator-dependent. It is widely used not only by radiologists but also by physicians with different levels of experience. The aim of this study was to investigate whether US with computer-aided diagnosis (CAD) has assisting roles to physicians in the diagnosis of thyroid nodules. 451 thyroid nodules evaluated by fine-needle aspiration cytology following surgery were included. 300 (66.5%) of them were diagnosed as malignancy. Physicians with US experience less than 1 year (inexperienced, n = 10), or more than 5 years (experienced, n = 3) reviewed the US images of thyroid nodules with or without CAD assistance. The diagnostic performance of CAD was comparable to that of the experienced group, and better than those of the inexperienced group. The AUC of the CAD for conventional PTC was higher than that for FTC and follicular variant PTC (0.925 vs. 0.499), independent of tumor size. CAD assistance significantly improved diagnostic performance in the inexperienced group, but not in the experienced groups. In conclusion, **t**he CAD system showed good performance in the diagnosis of conventional PTC. CAD assistance improved the diagnostic performance of less experienced physicians in US, especially in diagnosis of conventional PTC.

## Introduction

Ultrasonography (US) is the primary diagnostic tool used to access the malignancy risk of thyroid nodules. However, the accuracy of diagnostic US is highly operator-dependent and requires years of experience and training to read US image^1^. To optimize the use of diagnostic US for assessing the malignancy risk of thyroid nodules, various US risk stratification systems have been developed by several societies, such as the American College of Radiology (ACR)^2^, the American Thyroid Association^3^, the European Thyroid Association^4^, the Korean Society of Thyroid Radiology^5^, and the American Association of Clinical Endocrinologists, American College of Endocrinology, and Associazione Medici Endocrinologi^6^. Nonetheless, these US risk stratification systems have shown differences in diagnostic performance depending on the study group^7–9^, and inter-observer variability for thyroid US can be high even when a single risk stratification system is used^10^.

Computer-aided diagnosis (CAD) systems have been developed and applied for US diagnostics in various medical fields, catching up with the rapidly developing techniques of machine learning. Several recent studies showed that the diagnostic performance of machine learning in US CAD systems was comparable to that of expert radiologists^11–15^. However, a meta-analysis including 4 studies from the Samsung CAD system and 1 study from independently developed CAD system from China demonstrated that the specificity and the diagnostic odds ratio of the CAD system were lower than those of the experienced radiologist, while the sensitivity of the CAD system was similar^16^. We recently developed another US CAD system for thyroid nodule diagnosis using a machine learning method involving a deep convolutional neural network (CNN) model^17^. This system showed comparable or higher diagnostic performance than that of expert radiologists, however further validation of its diagnostic performance in various clinical settings and exploration of appropriate clinical use is needed.

Thyroid nodules are a common medical problem, and US is widely employed in the diagnosis of thyroid nodules not only by expert radiologists in the hospital but also by physicians in the primary clinics. However, weather the US CAD system is beneficial to the less experienced physicians or in the primary care setting has not been fully studied yet. The aim of this study was to investigate the potential benefits of the US CAD system in the diagnosis of thyroid nodules for less experienced physicians.

## Results

### Clinical characteristics of thyroid nodules

The clinical characteristics of the thyroid nodules are presented in Table 1. Of the 451 enrolled thyroid nodules, 300 nodules (66.5%) were surgically confirmed as malignant. Compared to the benign nodules, the malignant nodules were more frequently found in male patients (29.3% vs. 15.2%, *p* = 0.001) and were smaller on average (1.81 ± 1.0 vs. 2.52 ± 1.2 cm, *p* < 0.001). Patients’ mean age at the time of diagnosis was similar between groups. The cases of thyroid cancer were categorized as conventional papillary thyroid carcinoma (cPTC), follicular variant papillary thyroid carcinoma (fvPTC), follicular thyroid carcinoma (FTC), medullary thyroid carcinoma, poorly differentiated thyroid carcinoma, and anaplastic thyroid carcinoma. cPTC, fvPTC, and FTC accounted for 83.7%, 7.0%, and 7.3% of the malignant nodules, respectively. The tumor size was < 2 cm in 78.9% of cPTCs, while 65.1% of FTCs and fvPTCs combined (FTC/fvPTC) had a size of ≥ 2 cm (*p* < 0.001, Supplementary Table S1). Of the benign nodules, 38.4% were follicular adenoma, 31.8% were nodular hyperplasia, 23.8% were NIFTP, and 6.0% were other benign lesions.

**Table 1:** Baseline characteristics of thyroid nodules.

	Total	Benign	Malignancy	*P-*value
N (%)	451	151 (33.5)	300 (66.5)	
Age of diagnosis, yrs	50.0 ± 14.3	51.7 ± 13.5	49.1 ± 14.6	0.064
Male sex, n (%)	112 (24.8)	23 (15.2)	89 (29.3)	0.001
Size, cm	2.05 ± 1.1	2.52 ± 1.2	1.81 ± 1.0	< 0.001
**Histologic subtype, n (%)**				
cPTC	–	–	251 (83.7)	
fvPTC	–	–	21 (7.0)	
FTC	–	–	22 (7.3)	
MTC/PDTC/ATC	–	–	6 (2.0)	
Follicular adenoma	–	58 (38.4)	–	
Nodular hyperplasia	–	48 (31.8)	–	
NIFTP	–	36 (23.8)	–	
Other benign lesions	–	9 (6.0)	–	

cPTC, conventional papillary thyroid carcinoma; fvPTC, follicular variant papillary thyroid carcinoma; FTC, follicular thyroid carcinoma; MTC, medullary thyroid carcinoma; PDTC, *poorly differentiated thyroid carcinoma*; ATC, anaplastic thyroid carcinoma; NIFTP, noninvasive follicular thyroid neoplasm with papillary-like nuclear features, *p-*value for benign vs. malignancy.

### Diagnostic performance of thyroid US CAD

**Figure 1:**
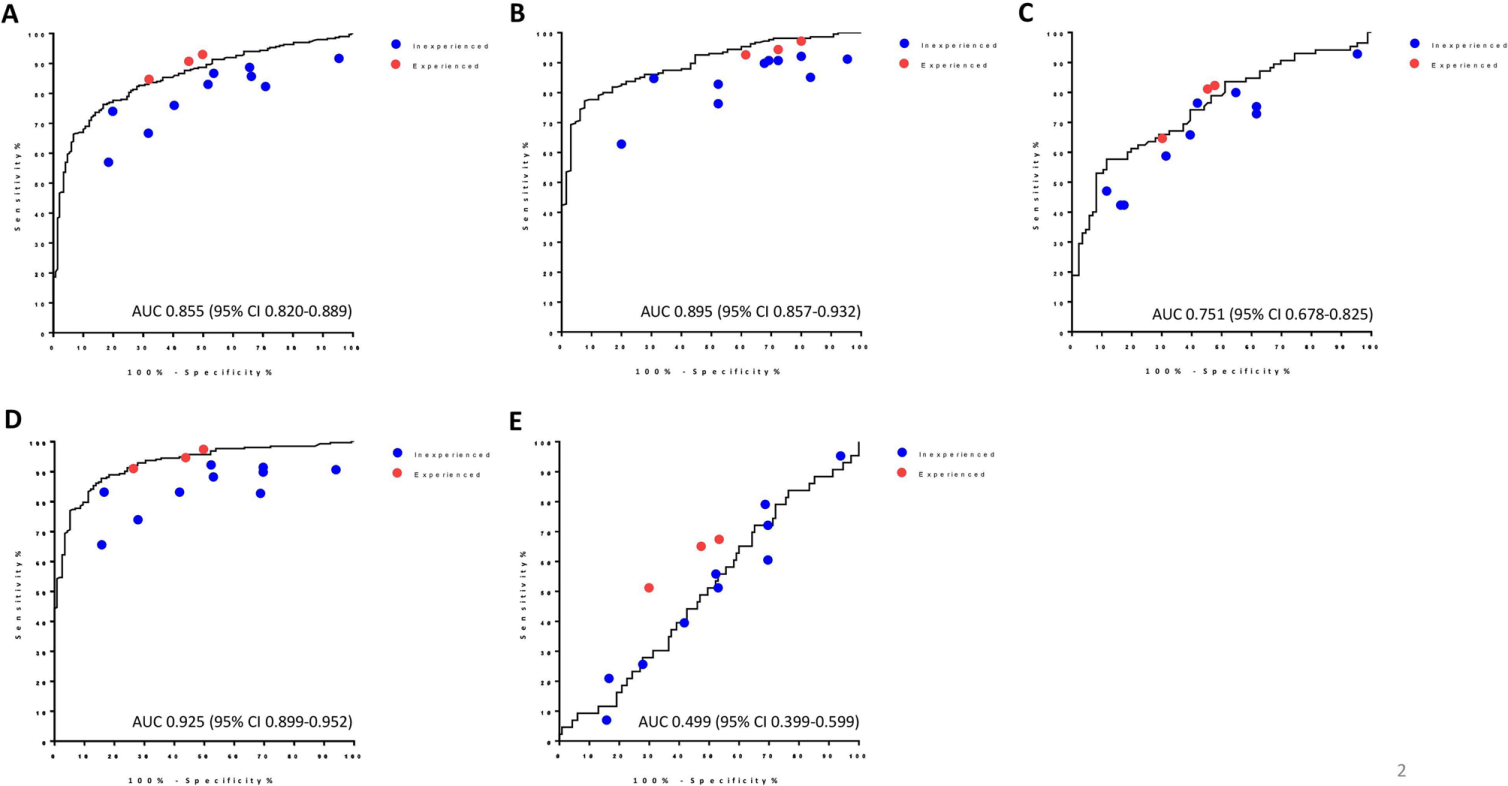
Comparison of diagnostic performance between CAD and physicians with different levels of experience. The ROC curves and AUC of CAD in the diagnosis of thyroid nodules are demonstrated in black solid lines in each graph for (**A**) all nodules, (**B**) nodules with a size < 2 cm, (**C**) nodules with a size ≥ 2 cm, (**D**) nodules diagnosed as cPTC, and (**E**) nodules diagnosed as FTC and fvPTC. Dots on each graph indicate the diagnostic performance (sensitivity and specificity) of the individual physicians in inexperienced (blue), and experienced (red) groups. CAD, computer-aided diagnosis; ROC, receiver operating characteristic curve; AUC, area under the curve; cPTC, conventional papillary thyroid carcinoma; FTC, follicular thyroid carcinoma; fvPTC, follicular variant papillary thyroid carcinoma.

**Figure 2:**
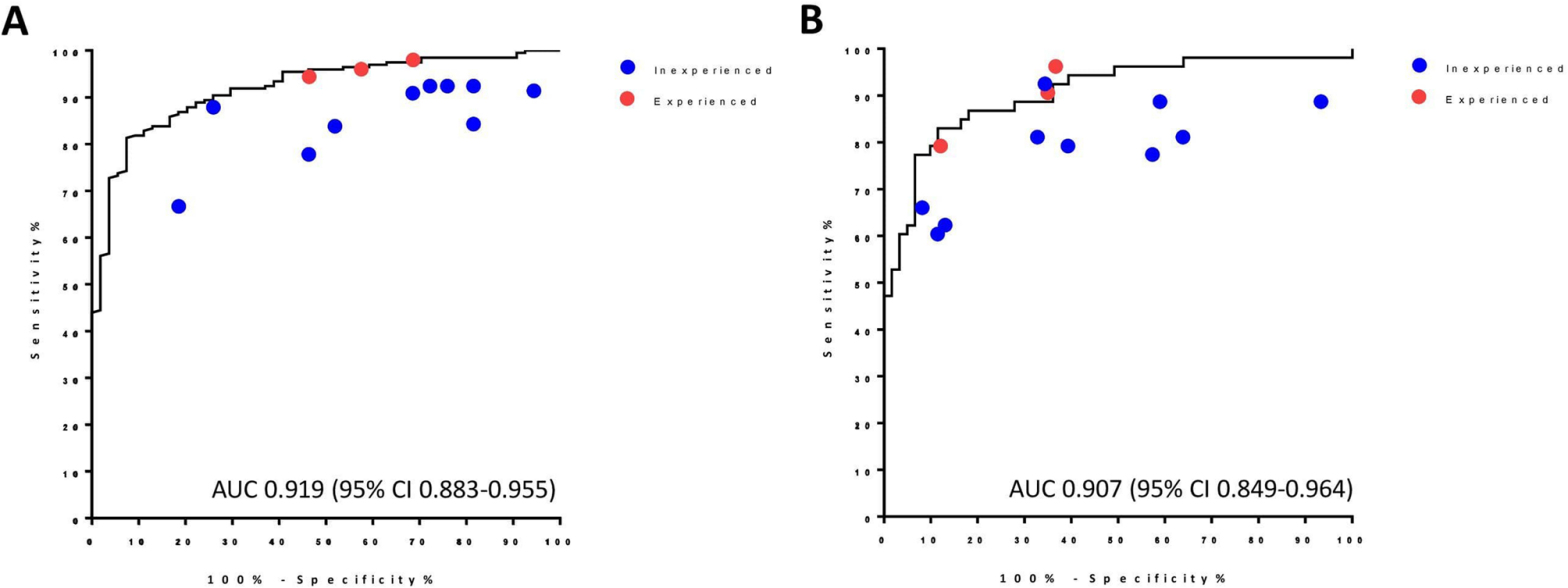
Diagnostic performance of CAD and physicians for cPTC according to nodule size. The ROC curves and AUC of CAD for cPTC are presented for thyroid nodule with (**A**) a size < 2 cm, and (**B**) a size ≥ 2 cm. Dots on each graph indicate the diagnostic performance (sensitivity and specificity) of the individual physicians in the inexperienced (blue), and experienced (red) groups. CAD, computer-aided diagnosis; ROC, receiver operating characteristic curve; AUC, area under the curve; cPTC, conventional papillary thyroid carcinoma.

The diagnostic performance of the CAD system is presented in Table 2 and Fig. 1. Overall, the AUC was 0.855 (Fig. 1A), and the sensitivity, specificity, PPV, and NPV, and accuracy were 85.3%, 63.6%, 82.3%, 68.6%, and 78.0%, respectively (Table 2). In the subgroup analysis, the CAD system showed higher diagnostic performance for thyroid nodules with a size < 2 cm than for larger nodules (≥ 2 cm) in terms of AUC (0.895 vs. 0.751, Fig. 1B,C), sensitivity (94.4% vs. 62.4%), PPV (84.9% vs. 73.6%), NPV (70.7% vs. 67.6%), and accuracy (82.9% vs. 70.2%). Since cPTC was significantly smaller than the other cancers (Supplementary Table S1), we then analyzed the diagnostic performance of the CAD system according to histologic subgroup. Compared to FTC/fvPTC, a higher AUC was found for cPTC (0.925 vs. 0.499, Fig. 1D,E). For cPTC, the CAD system also showed higher sensitivity (94.4% vs. 34.9%), PPV (85.3% vs. 26.8%), NPV (84.1% vs. 72.5%), and accuracy (85.0% vs. 56.3%). Interestingly, within the cPTC group, the diagnostic performance of the CAD system was similar regardless of size (AUC, 0.919 for nodules < 2 cm, Fig. 2A; 0.907 for nodules ≥ 2 cm, Fig. 2B).

**Table 2:** Diagnostic performance of computer-aided diagnosis (CAD).

	AUC	Sensitivity (%)	Specificity (%)	PPV (%)	NPV (%)	Accuracy (%)
Total	0.855 (0.820–0.889)	85.3 (0.822–0.881)	63.6 (57. 4–69.1)	82.3 (79.3–85.0)	68.6 (61.9–74.5)	78.0 (73.9–81.7)
**Size group**						
Size < 2 cm	0.895 (0.857–0.932)	94.4 (91.6–96.7)	44.6 (35.4–52.0)	84.9 (82.4–87.0)	70.7 (56.1–82.5)	82.9 (78.6–86.3)
Size ≥ 2 cm	0.751 (0.678–0.825)	62.4 (54.6–69.0)	77.9 (70.2–84.5)	73.6 (64.4–81.5)	67.7 (61.0–73.4)	70.2 (62.5–76.8)
**Histologic subtypes**						
cPTC vs. benign	0.925 (0.899–0.952)	94.4 (91.6- 96.6)	64.3 (58.1–69.0)	85.3 (82.7–87.2)	84.1 (76.0–90.2)	85.0 (81.1–87.9)
FTC and fvPTC vs. benign	0.499 (0.399–0.599)	34.9 (21.0–50.9)	64.3 (54.9–73.1)	26.8 (15.8–40.3)	72.5 (62.8–80.9)	56.3 (49.7–63.7)

Values (95% confidence intervals).

AUC, area under the curve; PPV, positive predictive value; NPV, negative predictive value; cPTC, conventional papillary thyroid carcinoma; FTC, follicular thyroid carcinoma; fvPTC, follicular variant papillary thyroid carcinoma.

### Diagnostic performance of physicians before and after CAD assistance

Next, the diagnostic performance was compared between the CAD system and physicians with different levels of experience, divided into the groups (the inexperienced and experienced groups) (Table 3). The inexperienced group showed significantly lower sensitivity (79.2% vs. 85.3%, *p* = 0.003), Specificity (48.6% vs. 63.6%, *p* < 0.001), PPV (76.6% vs. 82.3%, *p* = 0.009), NPV (52.0% vs. 68.6%, *p* < 0.001), and accuracy (68.9% vs. 78.0%, *p* < 0.001) than the CAD system. The experienced group showed higher sensitivity compared to that of the CAD system (89.4% vs. 85.3%, *p* = 0.017). CAD assistance significantly improved the diagnostic performance of the inexperienced group. The sensitivity and accuracy significantly improved after CAD assistance (sensitivity, 79.2% [before] vs. 84.5% [after], *p* = 0.010; accuracy, 68.9% [before] vs. 74.2% [after], *p* = 0.005, Table 3). Meanwhile, in experienced group, it cannot be said that there is a significant improvement after CAD assistance (Table 3).

**Table 3:** Diagnostic performance of physicians with different levels of experience before and after CAD assistance.

	CAD	Inexperienced				Experienced			
		Before (%)	After (%)	*P*^a^	*P*^b^	Before (%)	After (%)	*P*^a^	*P*^b^
Sensitivity	85.3	79.2	84.5	0.003	0.010	89.4	90.4	0.017	0.292
Specificity	63.6	48.6	53.8	< 0.001	0.103	57.6	58.3	0.079	0.463
PPV	82.3	76.6	79.5	0.009	–	80.7	81.1	0.261	–
NPV	68.6	52.0	65.9	< 0.001	–	73.1	75.2	0.133	–
Accuracy	78.0	68.9	74.2	< 0.001	0.005	77.4	79.6	0.396	0.134

ACR-TIRADS 4 was used as the cut-off to calculate the diagnostic performance of physicians. CAD, computer-aided diagnosis; Before, physicians before CAD assistance; After, physicians after CAD assistance. PPV, positive predictive value; NPV, negative predictive value.

*P*^a^, CAD vs. before; *P*^b^, before vs. after.

A subgroup analysis was performed according to the subtype of thyroid cancers. The AUC of the physicians was higher for PTC than for FTC/fvPTC (0.737–0.902 vs. 0.437–0.605), and CAD assistance significantly improved the AUC in most of the inexperienced group and a subset of experienced physicians for the diagnosis of cPTC, however, it cannot be said that there is a significant improvement with CAD assistance for the diagnosis of FTC/fvPTC (Supplementary Table S2). With CAD assistance, the mean sensitivity and accuracy for diagnosing cPTC significantly improved in the inexperienced group (sensitivity, 84.3% [before] vs. 91.0% [after], *p* = 0.001; accuracy, 73.2% [before] vs. 79.9% [after], *p* = 0.001), however, it cannot be said that there is a significant improvement in the experienced group (Table 4). Additionally, the mean accuracy for diagnosing cPTC significantly increased after CAD assistance, regardless of nodule size (nodules < 2 cm, 75.8% [before] vs. 81.7% [after], *p* = 0.011; nodules ≥ 2 cm, 67.5% [before] vs. 75.9% [after], *p* = 0.027) in the inexperienced group (Supplementary Table S3).

**Table 4:** Comparisons of diagnostic performances between CAD and physicians before and after CAD assistance according to the pathologic subtype.

	CAD	Inexperienced				Experienced			
		Before (%)	After (%)	*P*^a^	*P*^b^	Before	After	*P*^a^	*P*^b^
**cPTC**									
Sensitivity	94.4	84.3	91.0	< 0.001	0.001	94.6%	95.5%	0.564	0.243
Specificity	64.3	49.1	55.6	< 0.001	0.082	60.1	61.7	0.202	0.388
PPV	85.3	79.5	82.7	0.009	–	83.7	84.5	0.272	–
NPV	84.1	56.9	76.9	< 0.001	–	83.1	86.5	0.470	–
Accuracy	85.0	73.2	79.9	< 0.001	0.001	83.6	84.9	0.265	0.265
**FTC and fvPTC**									
Sensitivity	34.9	50.7	48.4	0.021	0.416	61.2%	62.0%	< 0.001	0.540
Specificity	64.3	49.1	55.6	< 0.001	0.099	56.5%	57.4%	0.053	0.460
PPV	26.8	26.3	28.8	0.518	–	34.2%	35.5%	0.152	–
NPV	72.5	73.5	76.0	0.451	–	79.2%	80.4%	0.066	–
Accuracy	56.3	49.6	53.6	0.055	0.162	57.6%	58.2%	0.403	0.469

ACR-TIRADS 4 was used as the cut-off to calculate the diagnostic performance of physicians.

CAD, computer-aided diagnosis; Before, physicians before CAD assistance; After, physicians after CAD assistance; PPV, positive predictive value; NPV, negative predictive value; cPTC, conventional papillary thyroid carcinoma; FTC, follicular thyroid carcinoma; fvPTC, follicular variant papillary thyroid carcinoma.

*P*^a^, CAD vs. before; *P*^b^, before vs. after.

## Discussion

In this study, the diagnostic performance for assessing the malignancy risk of thyroid nodules using US was compared between the CAD system and physicians with various levels of US experience, and the role of CAD assistance for physicians not board-certified radiologists was investigated. The AUC of the CAD system was 0.855 for all thyroid nodules and 0.925 for nodules diagnosed as cPTC, which was much higher than the AUC for nodules diagnosed as FTC/fvPTC. The diagnostic performance of physicians with less US experience was significantly lower than that of the CAD system, and CAD assistance improved their performance. Collectively, the present study demonstrated the beneficial role of assistance from the US CAD system for physicians with insufficient US training.

US is the most sensitive and widely used diagnostic tool for thyroid nodule assessment. Malignant nodules (especially PTCs) have specific US features in terms of echogenicity, solidity, orientation, and the presence of microcalcification^3,18^. Nonetheless, the reported diagnostic value of US varies considerably across studies, with high inter-performer and inter-observer variability. Although several guidelines have been established by related societies^2–6^, high inter-observer variability was still observed even among board-certified radiologists (κ = 0.51)^10^. In the real-world practice the diagnostic performance of US in accessing thyroid nodule showed big difference depending on the experience levels of operators. With this background, CAD systems have shown promise in overcoming these limitations of US performance, and robust technical developments have recently been achieved through breakthroughs in deep learning technology based on artificial neural networks^11,12,14,15^. Indeed, previous studies reported that CAD systems developed using deep learning models showed comparable diagnostic performance to expert radiologists, and the present study (AUC, 0.855) also showed similar performance to that reported in other studies (AUC, 0.87–0.947)^14,15,19^.

The present study demonstrated that a US CAD system established by a deep learning method (CNN) can furnish useful diagnostic assistance for less experienced physicians. For mimicking the real-world practice, this study recruited physicians not radiologists, and divided them into two groups according to their years of US experience. Although the number of physicians in the experienced group was small (n = 3), the diagnostic performance between the inexperienced and experienced group was significantly different.

Since US is widely used both by well-trained radiologists and by physicians in their general clinics, the present study stated the first step to verify the clinical use of the US CAD system. However, several points need to be considered regarding the application of the present CAD system in the practice of primary care physicians. In the development and validation process of the current CAD system, both the training set and the study set of nodules were enrolled from a tertiary referral hospital which are different from those of the primary care system. Furthermore, the enrolled nodules in the present study were all surgically diagnosed, which can lead to selection bias. Thus, further study is needed in primary care conditions.

In our daily practice, we generally use K-TIRAS system based on the short decision tree model, because it is easy and fast. However, to compare the diagnostic performance with or without CAD assistance we also used ACR-TIRADS which applies point-based system scoring system, scoring range from 0 to 14, since it showed the best sensitivity compared to other TIRADS^20^. Further study is needed to determine whether CAD-assistance can be widely applied in various TIRADS using field.

Additionally, the present study has several limitations. First, the US CAD system was originally developed using nodules 1 cm or larger, so it cannot be applied to nodules smaller than 1 cm. Although, the present study showed excellent results for the diagnosis of PTC using the CAD system in nodules of any size, an expanded CAD system would be needed for micro-nodules, which are identified at an increasing frequency. Second, the CAD system showed no beneficial role for the diagnosis of FTC/fvPTC. The AUC for FTC/fvPTC was 0.499, which was similar to that of physicians regardless of experience. Unlike cPTC, the US characteristics of FTC/fvPTC are very heterogeneous and non-specific^21–24^, and play a minimal role in the preoperative diagnosis^25^. Additionally, the US CAD system used in the present study was trained using PTC-dominant learning materials, as 96.5% of the nodule were PTCs. A challenge for further research would be to develop a highly advanced CAD system using artificial intelligence with sufficient data on FTC/fvPTCs.

In conclusion, the CAD system showed good diagnostic performance and had a beneficial assistive role for physicians with less US experience in assessing the malignancy risk of thyroid nodules, especially in PTCs. Therefore, this US CAD system can be a beneficial tool to assess less-experienced physicians in PTC-dominant areas.

## Materials and methods

### Study population

**Figure 3:**
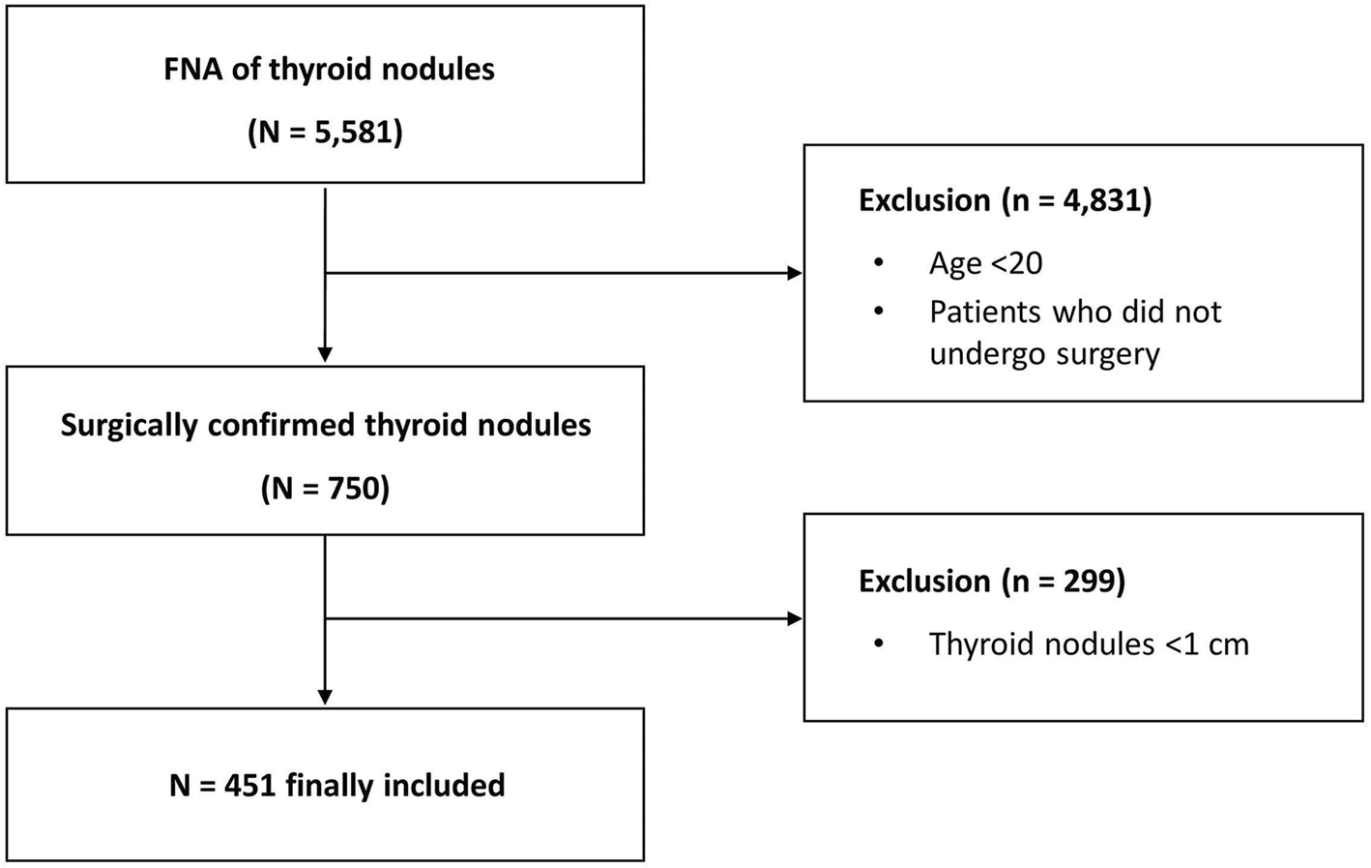
Flow diagram of study participants. FNA, fine-needle aspiration.

A total of 5581 US images of thyroid nodules from 4143 patients who had undergone fine-needle aspiration (FNA) at the Department of Endocrinology, Seoul National University Hospital from April 2014 to June 2019 were consecutively recruited and reviewed. The inclusion criteria were as follows: (i) patients ≥ 20 years of age, (ii) a maximal nodule diameter ≥ 1 cm, and (iii) patients whose nodules were pathologically confirmed by surgery. Finally, 451 thyroid nodules were enrolled (Fig. 3). Thirteen physicians, not board-certified radiologists, with various levels of US experience were recruited from three referral hospitals. Ten of them were general physicians who had US experience less than 1 year (designated as ‘inexperienced group’), and 3 of them were endocrine faculties with more than 5 years of experience in thyroid USG imaging and FNA procedures (designated as ‘experienced group’). This study was approved by the Institutional Review Board of Seoul National University Hospital (IRB No. 1911-039-1076)**.** Written informed consent has been obtained from each patient after full explanation of the purpose and nature of all procedures used. All methods were carried out in accordance with relevant guidelines and regulations.

### Cytologic and histologic evaluation of thyroid nodules

According to the recommendation of the Korean Thyroid Imaging Reporting and Data System (K-TIRADS)^5^, all nodules were evaluated by US and FNA was performed for suspicious nodules by experienced physicians. The cytology results were reported using the Bethesda System for Reporting Thyroid Cytopathology^26^ by an expert pathologist who had more than 10 years of experience at a tertiary hospital. Surgery was performed in patients with Bethesda cytology categories IV, V, and VI. Additionally, patients having nodules with Bethesda cytology categories II or III also underwent surgery if they have large nodule size, simultaneous presence of other nodules confirmed as malignancy, or the presence of compressive symptoms. Noninvasive follicular thyroid neoplasm with papillary-like nuclear features (NIFTP) was defined as a benign lesion.

### Ultrasonography examinations

US examinations were performed using high-resolution ultrasound machines (LOGIQ7; GE Healthcare, Milwaukee, WI, USA or Affinity 50G; Philips Healthcare, Bothell, WA, USA). Each system was equipped with a linear, high-frequency transducer (5–14 MHz). After screening patients, we selected the representative images of each thyroid nodules in which the elements constituting TIRADS (composition, echogenicity, shape, margin, echogenic foci) are clearly visible (Supplementary Fig. S1), and saved it as a JPEG file. A square region of interest for each nodule was drawn by an expert radiologist (J.Y.K). After the CAD system calculated the cancer probability, the US images of thyroid nodules were reviewed by 13 physicians.

The physicians reviewed the US images twice using the ACR-TIRADS^2^. First, the US images were provided for 30 s and the physicians scored it without CAD-assistance. Immediately after first scoring, the results of CAD system, representing dichotomized as cancer (1) or benign (0), were provided to the physicians. The physicians re-reviewed the same US image again for 30 s, and re-scored it. All physicians were blinded to the patients’ clinical information and pathology results.

### US CAD system

To evaluate malignancy risk, we used our CAD system that had been developed using a deep CNN model. The detailed development protocol of the US CAD system has been published previously^17^. Briefly, the algorithm was trained using 13,560 US images of thyroid nodules that were either surgically or cytologically proven as benign or malignant. For internal and external validation tests, surgically confirmed thyroid nodules were obtained from three tertiary hospitals and the tests verified that the diagnostic performance of the CAD system was comparable or higher than that of expert radiologists. Once a US image is input into the CAD system, the results are presented as cancer probabilities (%), and the images are also classified as malignant or benign, with a cut-off value of a 50% probability of malignancy.

### Statistical analysis

Data are described as the mean ± standard deviation for continuous variables, and as numbers (percentages) for categorical variables. To compare mean values between the two groups, the Student *t* test was used for continuous variables, and the chi-square test was used for categorical variables. To assess the diagnostic performance of the CAD system and physicians, receiver operating characteristic curves were constructed and the area under the curve (AUC) was calculated. The cut-off value of ACR-TIRADS was determined using the Youden index, and TR4 was used as the cut-off to calculate the sensitivity, specificity, positive predictive value (PPV), negative predictive value (NPV), and accuracy of physicians. To compare diagnostic performance between the CAD system and physicians, the mean accuracy, sensitivity, and specificity of physicians were compared to those of the CAD system using the binominal test. The statistical analysis was performed using STATA version 13.1 (StataCorp, College Station, TX, USA), and *p*-values < 0.05 were considered to indicate statistical significance for all tests.

## Supplementary Information

Supplementary Information.

## Data Availability

The data presented in this study are available on request from the corresponding author.
