# Author Correction: A beneficial role of computer-aided diagnosis system for less experienced physicians in the diagnosis of thyroid nodule on ultrasound

**DOI:** 10.1038/s41598-021-00821-6

**Published:** 2021-10-21

**Authors:** Sunyoung Kang, Eunjung Lee, Chae Won Chung, Han Na Jang, Joon Ho Moon, Yujin Shin, Kyuho Kim, Ying Li, Soo Myoung Shin, Yoo Hyung Kim, Seul Ki Kwon, Chang Ho Ahn, Kyong Yeun Jung, A. Ram Hong, Young Joo Park, Do Joon Park, Jin Young Kwak, Sun Wook Cho

**Affiliations:** 1 Department of Internal Medicine, Seoul National University College of Medicine, Seoul, Republic of Korea; 2 Department of Internal Medicine, Seoul National University Hospital, Seoul, Republic of Korea; 3 Department of Internal Medicine, Uijeongbu Eulji Medical Center, Eulji University School of Medicine, Uijeongbu-si, Republic of Korea; 4 School of Mathematics and Computing, Yonsei University, Seoul, Republic of Korea; 5 Department of Internal Medicine, Seoul National University Bundang Hospital, Seongnam, Republic of Korea; 6 Department of Internal Medicine, Nowon Eulji Medical Center, Eulji University School of Medicine, Seoul, Republic of Korea; 7 Department of Internal Medicine, Chonnam National University Medical School, Gwangju, South Korea; 8 Department of Molecular Medicine and Biopharmaceutical Sciences, Graduate School of Convergence Science and Technology, Seoul National University, Seoul, Republic of Korea; 9 Department of Radiology, Research Institute of Radiological Science, Yonsei University College of Medicine, Seoul, Republic of Korea

Correction to: *Scientific Reports* 10.1038/s41598-021-99983-6, published online 14 October 2021

The original version of this Article contained errors in the Affiliations.

Affiliation 2 was incorrectly given as ‘Department of Internal Medicine, Seoul National University Hospital, 101 Daehak-ro, Chongno-gu, Seoul 03080, Republic of Korea.’ The correct affiliation is listed below.

Department of Internal Medicine, Seoul National University Hospital, Seoul, Republic of Korea

Additionally, Affiliation 9 was incorrectly given as ‘Department of Radiology, Severance Hospital, Research Institute of Radiological Science, Yonsei University College of Medicine, 50-1 Yonsei-ro, Seodaemun-gu, Seoul 03722, Republic of Korea’. The correct affiliation is listed below.

Department of Radiology, Research Institute of Radiological Science, Yonsei University College of Medicine, Seoul, Republic of Korea

The original Article has been corrected.

