# Supplementary material for: A beneficial role of computer-aided diagnosis system for less experienced physicians in the diagnosis of thyroid nodule on ultrasound
Source: Sci Rep. 2021 Oct 14;11:20448. doi: 10.1038/s41598-021-99983-6 (PMC8516898; doi:10.1038/s41598-021-99983-6)
Supplement: Supplementary file 1 — Supplementary Information. [file 41598_2021_99983_MOESM1_ESM.docx]

**A beneficial role of Computer-Aided Diagnosis system for less experienced physicians in the diagnosis of thyroid nodule on Ultrasound**

Sunyoung Kang, MD^1,2,3^, Eunjung Lee, PhD^4^, Chae Won Chung, MD^1,2^, Han Na Jang, MD^1,2^, Joon Ho Moon, MD, PhD^5^, Yujin Shin, MD^1,5^, Kyuho Kim, MD, PhD^5^, Ying Li, MD^1^, Soo Myoung Shin, MD^1,2^, Yoo Hyung Kim, MD, PhD^2^, Seul Ki Kwon, MD^1,2,3^, Chang Ho Ahn, MD, PhD^1,5^, Kyong Yeun Jung, MD^6^, A Ram Hong, MD, PhD^7^, Young Joo Park, MD, PhD ^1,2,8^, Do Joon Park, MD, PhD^1,2^, Jin Young Kwak, MD, PhD^9*^, Sun Wook Cho, MD, PhD^1,2*^

^1^Department of Internal Medicine, Seoul National University College of Medicine, Seoul, Republic of Korea

^2^Department of Internal Medicine, Seoul National University Hospital, Seoul, Republic of Korea

^3^Department of Internal Medicine, Uijeongbu Eulji Medical Center, Eulji University School of Medicine, Uijeongbu-si, Republic of Korea

^4^School of Mathematics and Computing, Yonsei University, Seoul, Republic of Korea.

^5^Department of Internal Medicine, Seoul National University Bundang Hospital, Seongnam, Republic of Korea.

^6^Department of Internal Medicine, Nowon Eulji Medical Center, Eulji University School of Medicine, Seoul, Republic of Korea

^7^Department of Internal Medicine, Chonnam National University Medical School, Gwangju, South Korea

^8^Department of Molecular Medicine and Biopharmaceutical Sciences, Graduate School of Convergence Science and Technology, and College of Medicine or College of Pharmacy, Seoul National University

^9^Department of Radiology, Severance Hospital, Research Institute of Radiological Science, Yonsei University College of Medicine, Seoul, Republic of Korea

***Corresponding Author**

Sun Wook Cho, M.D., Ph.D.

Department of Internal Medicine, Seoul National University Hospital

101 Daehak-ro, Chongno-gu, Seoul, Republic of Korea, 03080

Tel: +82-2-2072-4761; Fax: +82-2-762-2292, E-mail address: swchomd@snu.ac.kr

Jin Young Kwak, M.D., Ph.D.

Department of Radiology, Severance Hospital

50-1 Yonsei-ro, Seodaemun-gu, Seoul, Republic of Korea, 03722

TEL: 82-2-2228-7400, FAX: 82-2-2227-8337, E-mail: [docjin@yuhs.ac](mailto:docjin@yuhs.ac)

**Short title:** Computer-Aided Diagnosis in Thyroid Nodules

**Keywords:** thyroid nodule, thyroid cancer, ultrasonography, computer-aided diagnosis, deep learning

Supplementary table S1. Tumor Sizes of Thyroid Cancers According to the Histologic Subtype

|  | Total | 1~2 cm | 2-4 cm | ≥4 cm | *P* |
| --- | --- | --- | --- | --- | --- |
| cPTC | 251 | 198 (78.9) | 51 (20.3) | 2 (0.8) | <0.001 |
| fvPTC | 21 | 10 (47.6) | 8 (38.1) | 3 (14.3) |  |
| FTC | 22 | 5 (22.7) | 7 (31.8) | 10 (45.5) |  |
| MTC/PDTC/ATC | 6 | 2 (33.3) | 3 (50.0) | 1 (16.7) |  |

Data are presented as number (%). cPTC, conventional papillary thyroid carcinoma; fvPTC, follicular variant papillary thyroid carcinoma; FTC, follicular thyroid carcinoma; MTC, medullary thyroid carcinoma; PDTC, poorly differentiated thyroid carcinoma; ATC, anaplastic thyroid carcinoma.

Supplementary Table S2. Comparisons of AUCs Between CAD and Physicians Before and After CAD Assistance According to the Pathologic Subtype.

| AUC | cPTC (n = 251) | | | |  | FTC and fvPTC (n = 43) | | | |
| --- | --- | --- | --- | --- | --- | --- | --- | --- | --- |
|  | CAD | Before | After | *P* |  | CAD | Before | After | *P* |
|  | 0.925 |  |  |  |  | 0.499 |  |  |  |
| E1 (0M) |  | 0.815***** | 0.864 | 0.009 |  |  | 0.520 | 0.539 | 0.616 |
| E2 (0M) |  | 0.761***** | 0.774 | 0.287 |  |  | 0.506 | 0.500 | 0.788 |
| E3 (0M) |  | 0.805***** | 0.864 | <0.001 |  |  | 0.501 | 0.520 | 0.613 |
| E4 (0M) |  | 0.815***** | 0.821 | 0.512 |  |  | 0.536 | 0.533 | 0.843 |
| E5 (0M) |  | 0.821***** | 0.847 | 0.008 |  |  | 0.566 | 0.599 | 0.167 |
| E6 (0M) |  | 0.778***** | 0.824 | <0.001 |  |  | 0.437 | 0.449 | 0.239 |
| E7 (0M) |  | 0.737***** | 0.800 | <0.001 |  |  | 0.532 | 0.480 | 0.167 |
| E8 (0M) |  | 0.744***** | 0.772 | 0.238 |  |  | 0.532 | 0.515 | 0.421 |
| E11 (1Y) |  | 0.854***** | 0.856 | 0.345 |  |  | 0.540 | 0.539 | 0.331 |
| E12 (1Y) |  | 0.843***** | 0.867 | 0.010 |  |  | 0.545 | 0.567 | 0.156 |
| E15 (>5Y) |  | 0.880***** | 0.895 | 0.023 |  |  | 0.567 | 0.548 | 0.448 |
| E16 (>5Y) |  | 0.873***** | 0.888 | 0.086 |  |  | 0.545 | 0.575 | 0.215 |
| E17 (>5Y) |  | 0.902 | 0.903 | 0.739 |  |  | 0.605 | 0.600 | 0.549 |

AUC, area under the curve; cPTC, conventional papillary thyroid carcinoma; FTC, follicular thyroid carcinoma; fvPTC, follicular variant papillary thyroid carcinoma; CAD, computer-aided diagnosis; Before, physicians before CAD assistance; After, physicians after CAD assistance.

E1-17, Physicians 1-17; M, month; Y, year. *P*, before vs. after CAD assistance. *, *P* < 0.05 compared to CAD.

Supplementary Table S3. Comparison of the Diagnostic Performance for PTCs in Different Size Groups Between CAD and Physicians Before and After CAD Assistance

|  | CAD | Inexperienced | | | |  | Experienced | | | |
| --- | --- | --- | --- | --- | --- | --- | --- | --- | --- | --- |
|  |  | Before | After | *P ^a^* | *P ^b^* |  | Before | After | *P ^a^* | *P ^b^* |
| 1~2 cm |  |  |  |  |  |  |  |  |  |  |
| Sensitivity | 96.5 | 86.0% | 92.7% | <0.001 | 0.001 |  | 96.1% | 96.3% | 0.490 | 0.480 |
| Specificity | 46.3 | 38.3% | 41.3% | 0.170 | 0.415 |  | 42.5% | 44.4 | 0.333 | 0.45 |
| PPV | 86.8 | 84.2% | 85.8% | 0.166 | ⎯ |  | 85.9 | 86.3 | 0.392 | ⎯ |
| NPV | 78.1 | 41.1% | 66.5% | <0.001 | ⎯ |  | 74.2% | 75.0 | 0.392 | ⎯ |
| Accuracy | 85.7 | 75.8% | 81.7% | <0.001 | 0.011 |  | 84.5% | 85.1% | 0.334 | 0.425 |
| ≥2 cm |  |  |  |  |  |  |  |  |  |  |
| Sensitivity | 86.8 | 77.7% | 85.3% | 0.041 | 0.080 |  | 88.7% | 89.0% | 0.392 | 0.535 |
| Specificity | 80.3 | 58.7% | 68.2% | <0.001 | 0.075 |  | 72.1% | 73.2% | 0.096 | 0.474 |
| PPV | 79.3 | 66.0% | 73.5% | 0.020 | ⎯ |  | 73.4% | 76.3% | 0.194 | ⎯ |
| NPV | 87.5 | 72.6% | 84.6% | 0.006 | ⎯ |  | 88.0% | 86.3% | 0.515 | ⎯ |
| Accuracy | 83.3 | 67.5% | 75.9% | <0.001 | 0.027 |  | 79.8% | 81.5% | 0.207 | 0.359 |

ACR-TIRADS 4 was used as the cut-off to calculate the diagnostic performance of physicians. CAD, computer-aided diagnosis; Before, physicians before CAD assistance; After, physicians after CAD assistance; PPV, positive predictive value; NPV, negative predictive value. *P ^a^,* CAD vs. before; *P ^b^*, before vs. after.


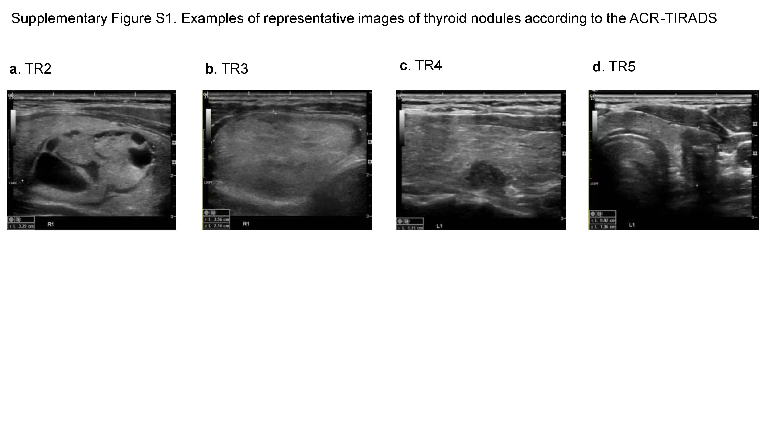


ACR-TIRADS, American College of Radiology- Thyroid Imaging Reporting and Data System; TR, TIRADS.
